# RT-PCR Assays for Seven Serotypes of Epizootic Haemorrhagic Disease Virus & Their Use to Type Strains from the Mediterranean Region and North America

**DOI:** 10.1371/journal.pone.0012782

**Published:** 2010-09-17

**Authors:** Narender S. Maan, Sushila Maan, Kyriaki Nomikou, Donna J. Johnson, Mehdi El Harrak, Hafsa Madani, Hagai Yadin, Serife Incoglu, Kadir Yesilbag, Andrew B. Allison, David E. Stallknecht, Carrie Batten, Simon J. Anthony, Peter P. C. Mertens

**Affiliations:** 1 Vector Borne Diseases Programme, Institute for Animal Health, Pirbright Laboratory, Woking, Surrey, United Kingdom; 2 United States Department of Agriculture (USDA) National Veterinary Services Laboratories, Ames, Iowa, United States of America; 3 Biopharma Laboratory, Rabbat, Akkari, Morocco; 4 Laboratoire Central Vétérinaire d'Alger, Hacen Badi, El Harrach, Alger, Algeria; 5 Kimron Veterinary Institute, Beit-Dagan, Israel; 6 Boronova Veteriner Konral de Aristirma, Izmir, Turkey; 7 Department of Virology, Uludag University Faculty of Veterinary Medicine, Gorukle, Bursa, Turkey; 8 Southeastern Cooperative Wildlife Disease Study, College of Veterinary Medicine, University of Georgia, Athens, Georgia, United States of America; 9 Department of Population Health, College of Veterinary Medicine, University of Georgia, Athens, Georgia, United States of America; IGMM CNRS 5535, France

## Abstract

Epizootic haemorrhagic disease virus (EHDV) infects wild ruminants, causing a frequently fatal haemorrhagic disease. However, it can also cause bluetongue-like disease in cattle, involving significant levels of morbidity and mortality, highlighting a need for more rapid and reliable diagnostic assays. EHDV outer-capsid protein VP2 (encoded by genome-segment 2 [Seg-2]) is highly variable and represents the primary target for neutralising antibodies generated by the mammalian host. Consequently VP2 is also the primary determinant of virus “serotype”, as identified in virus neutralisation tests (VNT). Although previous reports have indicated eight to ten EHDV serotypes, recent serological comparisons and molecular analyses of Seg-2 indicate only seven EHDV “types”. Oligonucleotide primers were developed targeting Seg-2, for use in conventional RT-PCR assays to detect and identify these seven types. These assays, which are more rapid and sensitive, still show complete agreement with VNT and were used to identify recent EHDV isolates from the Mediterranean region and North America.

## Introduction

*Epizootic haemorrhagic disease virus* (EHDV) is a distinct virus species within the genus *Orbivirus*, family *Reoviridae*, that is closely related to the ‘type species’ *Bluetongue virus* (BTV) [1], [2]. The EHDV particle has typical orbivirus morphology, consisting of an icosahedral virus-core (approximately 80 nm in diameter) composed of three concentric protein layers [1]–[4]. The inner ‘subcore’ layer, is composed of 120 copies of structural protein VP3 and surrounds three minor protein components, VPl, VP4 and VP6, as well as the ten linear dsRNA segments of the virus genome (Seg-1 to Seg-10) [5], [6]. The outer-core layer is composed of 780 copies of VP7 and provides a surface for attachment of the 60 trimers of VP2 and 180 trimers of VP5, which form the outer capsid layer [7]–[11]. Three different non-structural proteins, NS1, NS2, and NS3/NS3a, have also been identified in EHDV infected cells [1], [12], [13].

The current report by the International Committee for the Taxonomy of Viruses (ICTV) [2] indicates that there are eight serotypes of EHDV [2], which can be identified by the specificity of neutralising antibody interactions with the outer-surface of the virus (particularly with VP2) in ‘virus neutralisation tests’ (VNT) [14]–[16]. VP2 is the most variable of the EHDV proteins, showing sequence differences that correlate with both the serotype and geographic origin (topotype) of the virus lineage [2], [15], [17]. However, recent molecular and serological data presented by Anthony et al [15] showed that the EHDV isolate from Nigeria (NIG1967/01) that is used at Institute for Animal Health (IAH) as a reference strain for EHDV-3, cross-neutralises in VNTs with the reference isolate of EHDV-1 (New Jersey - USA1955/01), indicating that they in fact belong to the same ‘serotype’. EHDV strain ‘318’, which was first isolated in Bahrain in 1983 (BAR1983/01) and subsequently recovered from sentinel calves in the Sudan [15], [18], [19] was (for both isolates) identified as a western strain of EHDV-6 (EHDV-6[W]). Consequently only seven distinct types of EHDV were identified [15].

Both EHDV-1 and EHDV-2 have been responsible for numerous large-scale epizootics in wild ungulates (especially white-tailed deer), sometimes with significant fatalities [20]. EHDV-1 (New Jersey strain- USA1955/01) was first described in the north-eastern USA [21]. EHDV-2 (represented by the Alberta strain - CAN1962/01), was originally isolated in southern Alberta, Canada, in 1962 [22] but is also known to circulate in both Australia and Japan (Ibaraki virus - JAP1959/01) [23], [24]. EHDV serotypes 3 and 4 had only ever been reported in Africa [25] (although there is now evidence that EHDV-3 is the same type as EHDV-1 – [15]). EHDV serotypes 5, 6 (represented by the prototype strain CSIRO 753), 7 and 8 were originally identified in Australia [16], [23]. Initially only EHDV-1 and 2 were identified on more than one continent. However, EHDV-6 has recently been identified in both the Middle East (strain 318) and the USA [17], and EHDV-7 was identified in Israel during 2006, showing that other EHDV serotypes also exist in widely separated geographic locations.

Under field conditions, EHDV can infect several different ruminant species, although clinical signs occur most often in wild ungulates (including white-tailed deer, mule deer and antelope). In these species the infection can be very debilitating and is often fatal [22], [26], [27]. Although EHDV infection is generally inapparent or very mild in livestock species [28], more severe ‘bluetongue-like’ disease was observed in cattle in Japan during 1959 [17], [29]–[32] and more recently in Israel, Morocco and Turkey during (2006–2007) [33]–[35].

Since 1998, multiple different BTV types have caused widespread disease outbreaks in Europe [36]–[40]. These events, which demonstrate the presence of vector-competent *Culicoides* species across the entire region, have been linked to climate change [41]. During the same period, multiple, previously exotic BTV types, were also isolated in the southern states of the USA [42]. The primary involvement of EHDV with wild rather than domesticated species, and a lack of rapid/reliable molecular assays, has lead to lack of information concerning the prevalence and distribution of different EHDV types, particularly in the Middle East and the Indian sub-continent. However, it is clear that EHDV has recently also expanded its geographic distribution and economic importance, with outbreaks in livestock in Israel, Morocco, Algeria, Turkey, the USA and Reunion Island during 2004–2009 [34], [35], [43]–[54]. These events suggest that EHD represents an increasing threat to southern Europe and the USA, and are reflected by its declaration as a notifiable disease by OIE [55].

Conventional procedures for the detection and typing of EHDV involve virus isolation in embryonated chicken eggs (ECE) or sheep, and/or adaptation to cell culture, followed by ‘typing’ using reference antiserum against the established serotypes in ‘virus neutralisation tests’ (VNT) [56]. Alternatively antiserum from infected animals can also be tested for its ability to neutralise reference strains of each virus serotype in ‘serum neutralisation tests’ (SNT) [57]. However, these procedures are costly, time consuming and require access to reference viruses and/or antisera. Experience with BTV suggests that they are also prone to cross-reactions between types, particularly in areas where multiple virus serotypes are co-circulating [58], [59]. RT-PCR based molecular assays have recently been developed for individual BTV types [60]–[63] that are more rapid and more reliable than these conventional serological methods and have become standard diagnostic tests in many BTV reference laboratories. However, to improve EHDV surveillance, there is a need for similar molecular assays to identify and ‘type’ EHDV infections.

Detection of EHDV has previously been reported using either real time or conventional RT-PCR assays, containing virus-species/serogroup specific primers and probes targeting conserved genome segments 5, 7 and/or 10 (NS1, VP7 and/or NS3 genes respectively) [14], [64]–[68]. This report describes the development of a new panel of EHDV ‘type-specific’ primers targeting genome segment 2 (Seg-2 - the VP2 gene), and their use to type and characterise EHDV strains involved in recent disease outbreaks in the Mediterranean region and North America.

## Materials and Methods

### Field isolates

The different EHDV and other orbivirus isolates (listed in Table 1 and 2) were grown in BHK-21 cell monolayers, until 100% cytopathic effect (CPE) was observed. Each isolate is stored in the IAH ‘dsRNA virus reference collection’ (dsRNA-VRC) with a collection number composed of ‘country code, year, and the number of the isolate in that year from that country [48], [49]. The samples used in these studies were taken from naturally infected animals in the field, by qualified veterinarians, as part of normal veterinary care and diagnostic testing procedures in the respective countries.

**Table 1:** List of EHDV isolates used in this study.

IAH-P* dsRNA virus reference collection number	Accession numbers for genome Seg-2	EHDV Serotype** (strain)	Location (year)
**Reference strains of EHDV (Eastern [E]/Western [W])**			
AUS1995/02[E]	HM156728	1(DPP2209 (95/724 #12))	Australia (1995)
USA1955/01[W]	AM744978	1 (New Jersey)	USA (1955)
NIG1967/01	AM745008	1 (Ib Ar 22619)	Nigeria (1967)
JAP1959/01[E]	AM745078	2 (Ibaraki virus)	Japan (1959)
AUS1979/05	AM744988	2 (CSIRO 439)	Australia (1979)
CAN1962/01[W]	AM744998	2 (Alberta)	Canada (1962)
JAP----/08	–	2 (Ibaraki virus)	Japan
NIG1968/01[W]	AM745018	4 (Ib Ar 33853)	Nigeria (1968)
AUS1977/01[E]	AM745028	5 (CSIRO 157)	Australia (1977)
AUS1981/07[E]	AM745038	6 (CSIRO 753)	Australia (1981)
BAR1983/01[W]	AM745068	6 (strain 318)	Bahrain (1983)
AUS1981/06[E]	AM745048	7 (CSIRO 775)	Australia (1981)
ISR2006/13[W]	HM156731	6 [W] & 7[W]***	Israel, Jordan Valley (2006)
AUS1982/06[E]	AM745058	8 (DPP59)	Australia (1982)
**Other field strains of EHDV**			
ALG2006/02	HM156729	6[W]	Medea, Algeria (2006)
MOR2006/05	HM156730	6[W]	Ahfir, Morocco (2006)
ISR2006/01	–	7[W]	Israel, Jordan Valley (2006)
ISR2006/02	–	7[W]	Israel, Jordan Valley (2006)
ISR2006/03	–	7[W]	Israel, Jordan Valley (2006)
ISR2006/04	–	7[W]	Israel, Jordan Valley (2006)
ISR2006/05	–	7[W]	Israel, Jordan Valley (2006)
ISR2006/06	–	7[W]	Israel, Jordan Valley (2006)
ISR2006/07	–	7[W]	Israel, Jordan Valley (2006)
ISR2006/08	–	7[W]	Israel, Jordan Valley (2006)
ISR2006/09	–	7[W]	Israel, Jordan Valley (2006)
USA2006/05	–	6[E]	Iroquois County IL, USA (2006)
USA2006/06	–	6[E]	Vermillion County IN, USA (2006)
USA2006/07	–	6[E]	Henry County IN, USA (2006)
USA2006/08	–	6[E]	Iroquois County IL, USA (2006)
USA2006/09	–	6[E]	Iroquois County IL, USA (2006)
TUR2007/01	–	6[W]	Mugla Turkey (2007)
TUR2007/02	–	6[W]	Mugla Turkey (2007)
TUR2007/03	–	6[W]	Mugla Turkey (2007)
TUR2007/04	–	6[W]	Mugla Turkey (2007)
TUR2007/05	–	6[W]	Mugla Turkey (2007)
TUR2007/06	–	6[W]	Mugla Turkey (2007)
TUR2007/07	–	6[W]	Fethiye- Mugla Turkey (2007)

Full details of these isolates are presented in [15], and are also available on the dsRNA virus reference collection website [48].

*Institute for Animal Health, Pirbright.

**Serotypes listed are as reclassified by Anthony et al [15], with NIG1967/01 as serotype 1, not serotype 3. They have also characterised the unclassified strain ‘318’ as a western topotype of serotype 6 (EHDV-6[W]).

***The only available sequence for a western strain of EHDV-7 was derived from a mixed virus isolate.

**Table 2:** List of other orbiviruses used in this study.

Virus	Strain	IAH-P* dsRNA virus reference collection number
Bluetongue virus (BTV)	1–24 Reference strains from South Africa	RSArrrr/01 – RSArrrr/24
African horse sickness virus (AHSV)	1–9 Reference strains from South Africa	RSArrah/01 - RSArrah/08 and PAKrrah/09
Equine encephalosis virus (EEV)	1–6 Reference strains from South Africa	RSA1976/01; RSA1967/01; RSA1971/04; RSA1974/04; RSA1974/01; RSA1991/01
Andasibe virus	–	MAD1979/01
Chobar Gorge virus	Chobar Gorge	NEP1970/01
Japanaut virus	–	–
Eubenangee virus (EUBV)	–	AUS1978/03
Matucare virus	–	–
Tembe virus	–	BRA1963/01
Tracambe virus	–	BRA1985/01
Palyam virus (PALV)	Sudan (SU3843)	SUD1982/03

Full details of these isolates are available on the dsRNA website [49].

*Institute for Animal Health, Pirbright.

#### Moroccan and Algerian EHDV isolates 2006

During 2006 an outbreak of EHD occurred in cattle near Ahfir in the East of Morocco. The virus was isolated in embryonated chicken eggs (ECE) at Biopharma Laboratory in Rabbat, Morocco, followed by passage in BSR cells, a clone of BHK-21 cells (designated isolate 2-451- passage 1E3BSR). A sample of the virus was sent to IAH Pirbright, passaged twice in BHK-21 cells, and then stored as isolate MOR2006/05. EHDV ALG2006/02 was isolated in BHK-21 cells (at IAH), from a lymph sample taken from a cow showing clinical signs of disease, during August 2006 in Medea, Algeria.

#### Israeli EHDV isolates 2006

Ten isolates were made in ECE at the Kimron Institute in Israel, from the blood of cows showing clinical signs of disease in the Jordan Valley. These isolates, which were provisionally identified as EHDV, were sent to IAH Pirbright and passaged once in BHK-21 cells, then stored in the dsRNA-VRC as isolates ISR2006/01 to ISR2006/09 and ISR2006/13.

#### American EHDV isolates 2006

In September, 2006, viruses were isolated by inoculation of cattle pulmonary artery endothelial (CPAE) cells cultures from tissues of a captive white-tailed deer from Iroquois County, Illinois, (Sample 06-473906-1 [dsRNA-VRC number USA2006/05]) and a wild deer from Vermillion County, Indiana (Sample 06-473906-2 [USA2006/06/]) [51], [52]. Four additional isolates from captive deer including one from Henry County, Indiana (Sample 06-473906-3 [dsRNA-VRC number USA2006/07]), and two from Iroquois County, Illinois (06-473906-5 - USA2006/08 and 06-473906-6 - USA2006/09) were confirmed as EHDV at the Southeastern Cooperative Wildlife Disease Study (SCWDS). Although these isolates tested positive for EHDV by RT-PCR, virus neutralisation techniques failed to detect either EHDV-1 or EHDV-2 (the types previously identified in the USA) at the SCWDS. The virus isolates were subsequently sent to the USDA National Veterinary Services Laboratories (NVSL) for additional VNT testing. The isolates were then sent to IAH, Pirbright, for serotype identification and confirmation, where they were grown in BHK-21 cells and stored in the dsRNA-VRC as mentioned above.

#### Turkish EHDV isolates 2007

Samples of ‘buffy coat’ were collected from six cows that were thought to be infected with EHDV, in the Mugla region of Turkey, during June/July of 2007. These were used to inoculate KC cells (*Culicoides sonorensis* cell line) at IAH Pirbright, generating virus isolates TUR2007/01 to TUR2007/06.

A sample of bovine organ homogenate (lung and spleen), taken on 26^th^ June 2007 from the Fethiye-Mugla region of Turkey, was also sent to IAH Pirbright and used to infect KC cells, generating isolate TUR2007/07. This was identified as EHDV using a serogroup specific real time RT-PCR targeting Seg-9, as described by the manufacturer (Laboratoire Service International - LSI).

### Extraction of Viral dsRNA

The dsRNA extracted either from EHDV infected cell pellets using the Trizol® (Invitrogen) [69], or from cell-free supernatant fluid, using the QIAamp Viral RNA Mini Kit from Qiagen according to manufacturer's instructions. Each isolate was processed separately to minimize the risk of sample-to-sample contamination.

### Selection of serotype-specific oligonucleotide primers

Comparisons of full-length VP2 sequences already obtained for strains of the different EHDV serotypes [14], [15] (listed in Table 1), as well as those generated during these studies (EHDV-1/AUS1995/02 [Ac. No. HM156728]; EHDV-6/ALG2006/02 [Ac. No, HM156729]; EHDV-6/MOR2006/05 [Ac. No.HM156730]; EHDV-7/ISR2006/13 [Ac. No. HM156731]) facilitated the identification of unique regions (showing intra-typic conservation and hetero-typic variation) in the Seg-2, for the design of serotype-specific RT-PCR primers. Wherever possible, these analyses included data for multiple isolates of each type, from different geographical origins, to help ensure that the resulting primers would amplify Seg-2 from different topotypes within the same serotype.

At least two primer-pairs were designed for each serotype (Table S1 – provided as online supporting information file). Primer-pairs (identified by the letter ‘A’, indicating ‘all’) were designed to detect any isolate of each EHDV serotype. Other primers were designed targeting Seg-2 of either eastern or western topotypes of each type (identified by the letters ‘E’ and ‘W’, respectively; Table S1 – supporting information). Individual primers were named as follows: ‘EHDV’ representing Epizootic haemorrhagic disease virus, followed by a number indicating the serotype; ‘S2’ to indicate they target Seg-2; ‘F’ representing forward, or ‘R’ representing reverse primers; and a number corresponding to the nucleotide binding position of the primer in Seg-2.

### Reverse transcription and amplification of Seg-2

Complementary DNA (cDNA) copies were synthesised (reverse transcribed) from denatured dsRNA templates, then amplified by a ‘single-tube’ method, using the One-Step RT-PCR kit (Qiagen, Courtaboeuf, France), using conditions described for amplification of cDNAs from Seg-2 of bluetongue virus (BTV) by Mertens et al [63]. Alternatively a single-tube reaction contained the SuperScript™ III one-step RT-PCR system (Invitrogen) and high fidelity platinum® Taq was used with RNA templates extracted from blood or cell culture supernatant, as described by Maan et al [37]. In some cases, full length cDNA copies of EHDV Seg-2 were also synthesised and sequenced, as described by Maan et al [70].

### Sequencing of Seg-2 amplicons

For confirmation of RT-PCR typing results, amplified cDNA products from Seg-2 were gel-purified and sequenced directly on an Applied Biosystems BigDye ddNTP 3730 capillary sequencer, using the PCR primers, as sequencing primers. The resultant sequences were analysed and aligned using the Clustal W program [71] and MAFFT (www.ebi.ac.uk/Tools/mafft/) and then subjected to either BLAST analysis and/or *in silico* comparisons to Seg-2 sequence data available for the 7 EHDV serotypes (including both western and eastern strains). Phylogenetic comparisons were made using Neighbour-Joining (NJ) algorithms and the trees were constructed in MEGA ver. 4.1 software [72], using nucleotide sequences in a pair-wise deletion, p-distance algorithm, and bootstrapped using 500 replicates.

## Results

### Development of RT-PCR assays and their evaluation for type specificity

**Figure 1:**
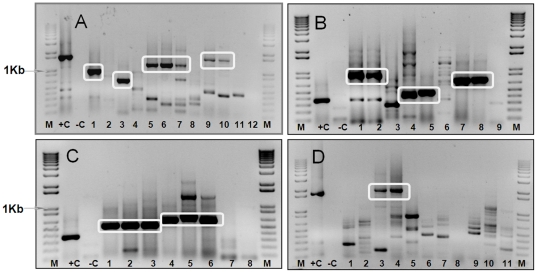
‘Type-specific’ primers for Seg-2 of EHDV-1 and EHDV-2. The cDNAs amplified from Seg-2 of EHDV-1 or EHDV-2 using type-specific primers, were analyzed by 1% agarose-gel-electrophoresis (AGE). **Panel A:** Lanes 1, 3, 5 and 9 show products from EHDV-1/AUS1995/02 using primer-pairs ‘1E1’ (918 bp), ‘1E2’ (660 bp), ‘1A1’ (1199 bp) and ‘1A2’ (1500 bp) respectively. Lanes 6 and 10: EHDV-1/USA1955/01, using primer-pairs ‘1A1’ and ‘1A2’. Lanes 7 and 11: EHDV-1/NIG1967/01, using primer-pairs ‘1A1’ and ‘1A2’. Lanes 2, 4, 8 and 12: Primer-pairs ‘1E1’, ‘1E2’, ‘1A1’ and ‘1A2’ amplified only small amounts of ‘incorrectly’ sized products from EHDV-6/BAR1983/01 (heterologous-control). Lane +C: BTV-6/RSArrrr/06 with primer-pair BTV-6/2/301F & BTV-6/2/790R (1631 bp) (+ve control: Maan et al [37]). **Panel B:** Lanes 1, 4 and 7 and Lanes 2, 5 and 8: Amplicons from EHDV-1/USA1955/01 and EHDV-1/ NIG1967/01 (respectively), using primer-pairs ‘1W1’ (888 bp), ‘1W2’ (488 bp) and ‘1W3’(735 bp). Lanes 3, 6 and 9: Primer-pairs ‘1W1’, ‘1W2’ and ‘1W3’ (respectively) generated only small amounts of ‘incorrectly’ sized products from EHDV-6/BAR1983/01 (heterologous-control). **Panel C:** Primer-pairs ‘2A1’ (531 bp) and ‘2A2’ (612 bp) were used with EHDV-2/CAN1962/01(Lanes 1 and 4); EHDV-2/JAP1959/01 (Lanes 2 and 5); EHDV-2/AUS1979/05 (Lanes 3 and 6); and EHDV-7/AUS1981/06 (heterologous control - Lanes 7 and 8). No amplification was detected in the control reactions. **Panel D:** Lanes 3 and 4 show amplicons from EHDV-2/CAN1962/01 and EHDV-2/USA1996/03 (respectively - both western strains) using primer-pair ‘2W1’ (1942 bp). Lanes 1 and 2: amplicons from eastern-strains of EHDV-2 (JAP1959/01 and AUS1979/05). Lanes 5 to 11: EHDV-1/USA1955/01, EHDV-4/NIG1968/01, EHDV-5/AUS1977/01, EHDV-6/USA2006/05, EHDV-7/AUS1981/06, EHDV-7/ISR2006/13 and EHDV-8/AUS1982/06 (respectively - as heterologous controls). Although minor bands were generated from both eastern-strains and heterologous-controls they were ‘incorrect’ sizes. Lane +C: products from BTV-6/NET2008/04, using primer-pair BTV-6/2/301F & BTV-6/2/790R (1631 bp) (+ve control: Maan et al [37]). Lane –C: is a negative-control (water). For primer positions and sequences see Table S1.

**Figure 2:**
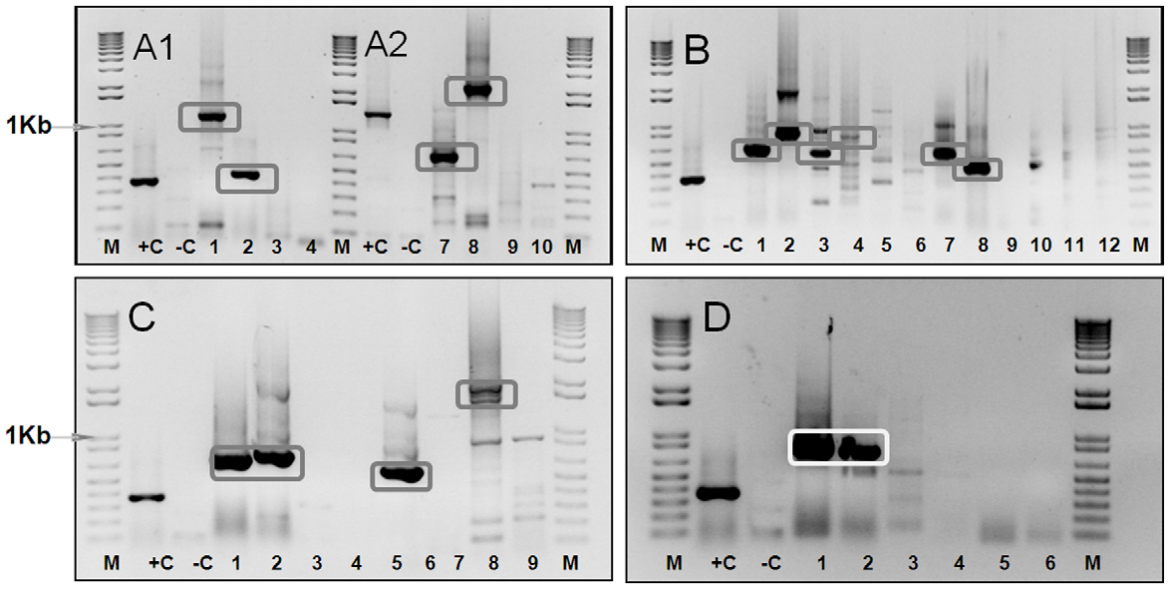
‘Type-specific’ primers for Seg-2 of EHDV-4, 5, 6, 7 and 8. cDNAs amplified from Seg-2 of EHDV-4 to 8 using type-specific primers, were analyzed by 1% agarose-gel-electrophoresis (AGE). **Panel A1:** Lanes 1 and 2: amplicons from EHDV-4/NIG1968/01, using primer-pairs ‘4W1’ (1161 bp) and ‘4W2’ (428 bp). Lanes 3 and 4: no products were generated from EHDV-5/AUS1977/01 (heterologous-control) using these primers. **Panel A2:** Lane 7 and 8: products from EHDV-5/AUS1977/01 using primer-pairs ‘5E1’ (680 bp) and ‘5E2’ (2199 bp). Lanes 9 and 10: no products were generated from EHDV-4/NIG1968/01 (heterologous-control) using these primer-pairs. Lane +C: products from BTV-1/RSArrrr/01, using primer-pair BTV-1/2/p524F & BTV-1/2/p968R (1334 bp) (+ve control: Mertens et al [63]). **Panel B:** Amplicons from EHDV-6 /AUS1981/07 (Lanes 1 and 2) and EHDV-6/BAR1983/01 (Lanes 3 and 4) using primer-pair ‘6A1’ (664 bp) and ‘6A2’ (859 bp). Lanes 5 and 6: smaller amounts of ‘incorrectly’ sized products from EHDV-8/AUS1982/06 (heterologous control) using the same primers. Lanes 7 and 8: amplicons from EHDV-6/BAR1983/01 using primer-pairs ‘6W1’ (573 bp) and ‘6W2’ (468 bp). Neither primer-pair amplified Seg-2 from EHDV-6/AUS1981/07 (eastern isolate - lanes 9 and 11), or EHDV-8/AUS1982/06 (heterologous-control - lanes 10 and 12). **Panel C:** Lanes 1 and 2: amplicons from EHDV-7/AUS1981/06 using primer-pairs ‘7E1’ (700 bp) and ‘7E2’ (730 bp). Lanes 5 and 8: products from EHDV-7/ISR2009/13 using primer-pairs ‘7W2’ (609 bp) and ‘7W1’ (1978 bp). Lanes 3, 4, 7 and 9: primer-pairs ‘7E1’, ‘7E2’, ‘7W2’ and ‘7W1’generated no amplification products from EHDV-2/AUS1979/05 (heterologous-control). **Panel D:** Lanes 1 and 2: amplicons from EHDV-8/AUS1982/06 using primer-pairs ‘8E1’ (803 bp) and ‘8E2’ (772 bp). No amplification was detected from EHDV-6/BAR1983/01 (Lanes 3, 4) or EHDV-6/AUS1981/07 (lanes 5 and 6) (heterologous-controls). Lane M: 1 Kb marker. Lane +C: BTV-9/RSArrrr/09 using primer-pair BTV-9/2/p377F & BTV-9/2/p485R (345 bp) (+ve control: Mertens et al [63]). Lane –C is a negative-control (water). For primer positions and sequences see Table S1.

Multiple primer-pairs were designed based on sequence data for Seg-2, targeting each of the seven EHDV serotypes (Table 1 and Table S1 - supporting information). These primers were tested in RT-PCR reactions containing RNA extracted from BHK-21 cells infected with the different EHDV serotypes (including both eastern and western topotypes, where available - Table 1). The resulting cDNAs were analysed by 1% agarose gel electrophoresis (AGE), to check both their sizes and relative level of synthesis. In each case, cDNAs of the expected size were generated from RNA belonging to the homologous EHDV serotype (Fig. 1 and 2). However, in most cases, significant amounts of cDNA of the expected size were only amplified in reactions containing RNA of the same topotype. None of the primer-pairs tested amplified cDNAs from RNA of even the most closely related of the heterologous EHDV serotypes, or from the other related orbiviruses tested (Table 1 and 2) (data not shown). In some serotypes it was possible to design primers using the combined sequences from both eastern and western strains within an individual serotype, which successfully amplified Seg-2 sequences from both major topotypes. These initial evaluations confirm *in silico* analyses, indicating that within the range of EHDV strains tested (Table 1 and 2) the primers listed in Table S1 (supporting information) are ‘type-specific’.

#### EHDV-1 (and 3)

Three primer-pairs (‘1W1’, ‘1W2’ and ‘1W3’) were designed, based on sequence data for Seg-2 of two western strains of EHDV-1 (USA1955/01 [Ac. No. AM744978] and NIG1967/01 [Ac. No. AM745008]). NIG1967/01 was previously classified as EHDV-3 but is now included in EHDV-1 [15]. Eastern topotype specific primer-pairs (‘1E1’ and ‘1E2’) were also designed and evaluated, using Seg-2 sequence data generated for EHDV-1/AUS1995/02 (Ac. No. HM156728). By combining Seg-2 sequence data from both eastern and western strains, two primer-pairs (‘1A1’ and ‘1A2’) were designed for amplification of cDNAs from Seg-2 of any of the three EHDV-1 isolates.

Primer-pairs ‘1E1’ and ‘1E2’ successfully amplified Seg-2 from the eastern strain of EHDV-1/AUS1995/02. Seg-2 of both eastern and western strains of EHDV-1 could be amplified using primer-pair ‘1A1’, while primer-pair ‘1A2’ amplified all strains of EHDV-1 with the exception of NIG1967/01 (regardless of topotype). None of the four pairs (‘1E1’, ‘1E2’, ‘1A1’ and 1A2’) showed cross-amplification with Seg-2 from reference strains of the remaining six EHDV serotypes, as shown for EHDV-6[W]/BAR1983/01 (Fig. 1 – Panel A). Both of the eastern strain-specific primer-pairs did not cross amplify when tested with the western strains of EHDV-1.

Although all three western strain-specific primer-pairs amplified cDNAs from several western isolates of EHDV-1 (including USA2001/01, USA2001/02, USA2001/03) the level of cDNA synthesis observed was significantly lower using primer-pair ‘1W1’ and RNA from NIG1967/01. No cross-amplification was detected using ‘1W1’, 1W2’ and ‘1W3’ with RNA from an eastern strain of EHDV-1/AUS1995/02 and other EHDV serotypes, as illustrated in Fig. 1 - Panel B for Seg-2 from BAR1983/01 (a western strain of EHDV-6).

#### EHDV-2

Two primer-pairs (‘2A1’ and ‘2A2’), were designed, based on sequence data for Seg-2 of two eastern strains (AUS1979/05 [Ac. No. AM744988] and JAP1959/01 [Ac. No. AM745078]) and one western strain (CAN1962/01 [Ac. No. AM744998]) of EHDV-2. These primers successfully amplified cDNAs from Seg-2 of all three EHDV-2 isolates, confirming their specificity for both eastern and western topotypes of EHDV-2 (Fig. 1 – Panel C). They also failed to cross-react with any of the other EHDV serotypes, as illustrated for isolate AUS1981/06 of EHDV-7 (the closest heterologous serotype to EHDV-2), confirming ‘type’-specificity (Fig. 1 - Panel C).

Sequence data for Seg-2 of the two eastern [E] and one western [W] strain of EHDV-2, were also used to design two sets of topotype specific primers, (‘2E1’ and 2W1’). The ‘2E1’ primer set successfully amplified Seg-2 from two EHDV-2[E] strains (AUS1979/05 and JAP1959/01) (data not show). Primer set ‘2W1’ also efficiently amplified cDNAs from two EHDV-2[W] strains (CAN1962/01 and USA1996/03; Fig. 1 – Panel D). Both ‘2E1’ and ‘2W1’ primer sets generated multiple cDNA bands from RNAs of heterologous EHDV serotypes (including isolate AUS1981/06 [EHDV-7] – Fig. 1 – Panel D for ‘2W1’), and from the heterologous topotype of EHDV-2, although none of these was the correct size. This confirms both the serotype and topotype specificity of primer sets ‘2E1’ and ‘2W1’ for EHDV-2.

#### EHDV-4 and EHDV-5

**Figure 3:**
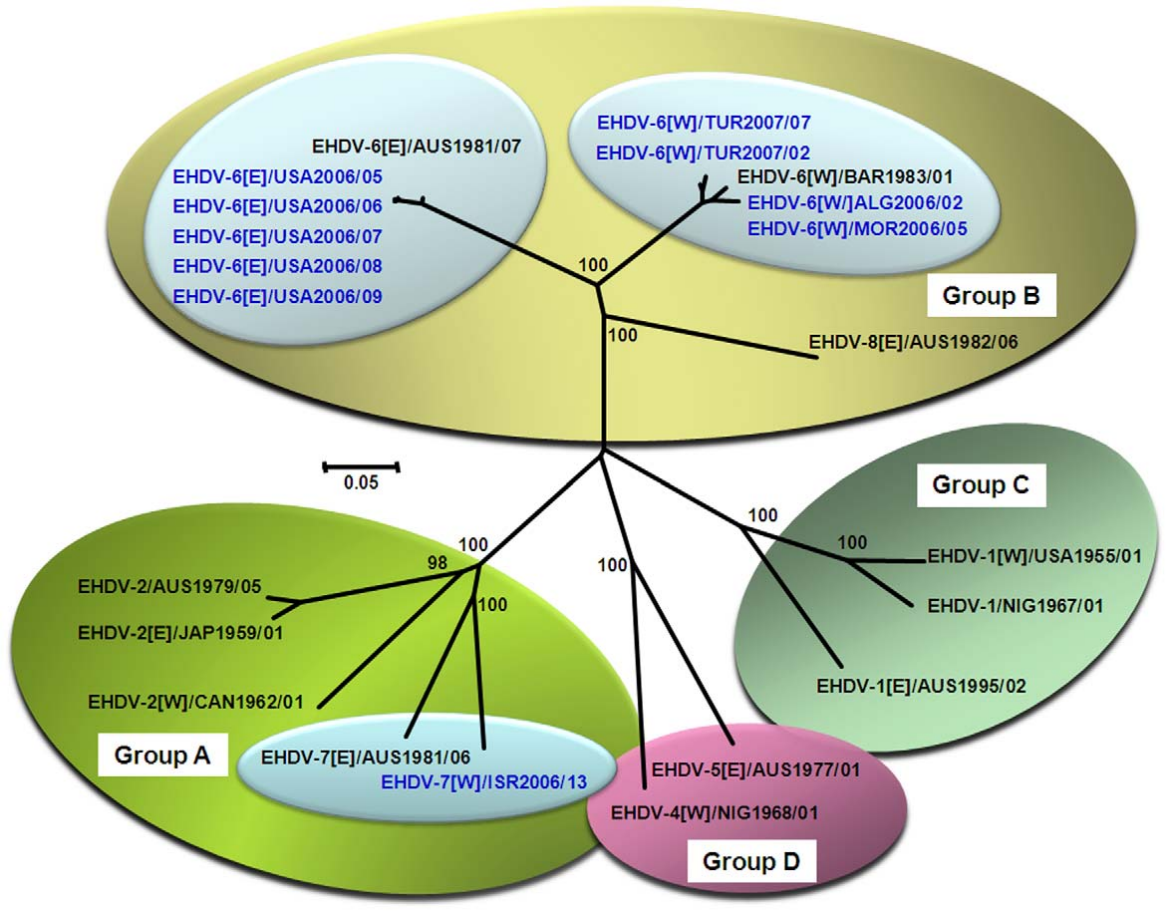
Neighbour-joining tree, showing relationships in Seg-2 between reference and field strains of EHDV. The tree was constructed using distance matrices, generated using the p-distance determination algorithm in MEGA 4.1 (500 bootstrap replicates) [72]. EHD viruses split into four distinct groups (A–D) based on Seg-2 sequences, reflecting serological relationships between virus strains [15]. Reference strains of EHDV are shown in black and field isolates are shown in blue font. Isolate designations: IAH ‘dsRNA virus reference collection number composed of ‘country code, year, and the number of the isolate in that year from that country [48], [49]. Full length Seg-2 sequences of Algerian and Moroccan 2006 strains were identical to each other and showed 97% nucleotide identity to the EHDV-6 [W]/BAR1983/01. ISR2006/13 showed 75% nucleotide identity to the EHDV-7[E]/AUS1981/06 in the upstream terminal 2867 base pairs of Seg-2 sequence. Seg-2 (328–947 nt) of American 2006 isolates were identical to each other and showed 97.7% identity to the EHDV-6 [E]/AUS1981/07. Seg-2 (120–684 nt) of Turkish 2007 isolates showed 98.9% identity to each other and showed 96.2–96.8% identity to the EHDV-6 [W]/BAR1983/01 Scale represents number of substitutions per site. Values at major branching points represent NJ bootstraps.

Two primers-pairs (‘4W1’ and ‘4W2’) were designed based on Seg-2 sequences from the western strain of EHDV-4 (NIG1968/01 [Ac. No. AM745018]), and two pairs (‘5E1’ and ‘5E2’) were based on an eastern strain of EHDV-5 (AUS1977/01 [Ac. No. AM745028]). Each primer set efficiently amplified cDNAs of the homologous virus strain and even though serotypes 4 and 5 are the most closely related serotypes to each other, no cross-reaction was detected between them (Fig. 2 - Panel A1 and A2 and Fig. 3). Evaluation of each primer-pair with the other heterologous EHDV serotypes (type 1, 2, 6, 7 and 8 – as listed in Table 1) also failed to amplify Seg-2, confirming ‘type specificity’ (data not shown).

#### EHDV-6

sequence data for Seg-2 of an eastern and a western isolate of EHDV-6, (AUS1981/07 [Ac. No. AM745038] and BAR1983/01 [Ac. No. AM745068], respectively) were used to design three sets of primers, one specific for the eastern topotype (‘6E1’) and two for the western topotype (‘6W1’ and ‘6W2’). Primer-pairs ‘6A1’ and ‘6A2’, were also designed from sequence data for an eastern strain of EHDV-6 (AUS1981/07 [Ac. No. AM745038]), amplified cDNAs from Seg-2 of both eastern and western EHDV-6 isolates, including the eastern reference strain of EHDV-6/AUS1981/07 as well as the eastern topotype of EHDV-6 from the USA (USA2006/05 – USA2006/09). Both primer-pairs (‘6A1’ and ‘6A2’) also generated cDNA bands of the correct size from BAR1983/01, belonging to the western topotype of EHDV-6 (Fig. 2 - Panel B).

In contrast primer-pairs ‘6E1’ as well as ‘6W1’ and ‘6W2’ only amplified Seg-2 from AUS1981/07 (EHDV-6[E]) or BAR1983/01 (EHDV-6[W]) respectively (Fig. 2 - Panel B – for ‘6W1’ and ‘6W2’). None of these EHDV-6 primer-pairs amplified cDNAs from Seg-2 belonging to any of the other EHDV serotypes (data not shown) including EHDV-8/AUS1982/06, the heterologous serotype that is most closely related to BTV-6 (Fig. 2 - Panel B).

#### EHDV-7

Sequence data for Seg-2 from an eastern and a western strain of EHDV-7 (AUS1981/06 [Ac. No. AM745048] and ISR2006/13 [Ac. No. HM156731] respectively) were used to design two primer-pairs for each topotype. Each primer-pair amplified cDNAs from Seg-2 of the homologous strain of EHDV-7 but no cross-topotype reaction was detected. No cross-amplification was detected using RNA templates from other EHDV reference strains belonging to serotype 1, 2, 4, 5, 6 and 8, as illustrated for isolate CAN1962/01 of EHDV-2 (the closest heterologous serotype to EHDV-7; Fig. 2 - Panel C). Our initial attempts to design primers that would detect Seg-2 of any isolate of EHDV-7 (regardless of topotype) were unsuccessful.

#### EHDV-8

Two primer-pairs (‘8E1’ and ‘8E2’) were designed based on the sequence of an eastern strain of EHDV-8 (AUS1982/06 [Ac. No. AM745058]). Both pairs successfully amplified cDNAs from Seg-2 of the homologous virus strain, but no cross-reaction was observed with eastern or western strains of EHDV-6 - the closest heterologous serotype to EHDV-8 (AUS1981/07 and BAR1983/01, respectively) (Fig. 2 - Panel D).

### Typing of North African field isolates of EHDV from 2006

RNA was extracted from BHK-21 cells infected with Algerian or Moroccan orbivirus isolates from 2006 (ALG2006/02 and MOR2006/05, respectively). These viruses were distinct from those used in the initial design of the EHDV ‘type-specific’ primers described above. The RNAs were initially tested using an EHDV ‘group-specific’ RT-PCR assay, containing primers targeting Seg-7 ([14], Table S1 - supporting information). In each case cDNA bands of the expected size were generated (results not shown) providing the initial identification of both isolates as EHDV. These RNA preparations were also tested using a commercially available EHDV ‘group-specific’ real-time RT-PCR (from LSI), giving cycle threshold (CT) values of 12 and 14 respectively, confirming their identity as EHDV.

ALG2006/02 and MOR2006/05 were subsequently tested by RT-PCR, using ‘type-specific’ primers listed in Table S1 (supporting information). cDNAs of the predicted sizes (573 and 468 base pairs [bps] respectively) were generated from either RNA preparation with primer-pairs ‘6W1’ and ‘6W2’. No cDNAs were generated with any of the other primer-pairs, including those directed against eastern strains of EHDV-6 (‘6E1’), providing the first indication that both isolates belong to the western topotype of EHDV-6 (EHDV-6[W]).

Full-length cDNA were also synthesised from Seg-2 of ALG2006/02 and MOR2006/05, using an ‘anchor-primer’ ligated to the RNA 3′ termini, then sequenced, as described by Maan et al [70]. The Seg-2 sequences generated were identical, reflecting the origin of both isolates from the same North African outbreak in 2006. These full length Seg-2 sequences were compared to EHDV strains belonging to each of the different serotypes (listed in Table 1), and both showed highest nucleotide (nt) identity (97%) to BAR1983/01, the isolate of EHDV-6[W] (previously identified as EHDV-318 – Fig. 3). This not only confirms that both virus isolates belong to EHDV-6[W], but also that primer-pairs ‘6W1’ and ‘6W2’ can be used to identify field isolates of this serotype and topotype. Subsequent comparisons to the reference strains of EHDV serotypes 1-8 [15] showed highest levels of identity (72.6% nt and 77% aa) to Seg-2 of EHDV-6[E] (AUS1981/07) and lowest levels (46% nt and 31% aa) to Seg-2 of EHDV-2/CAN1962/01.

Virus neutralisation tests (VNTs) of ALG2006/02 and MOR2006/05, showed high levels of neutralisation with reference antisera against BAR1983/01 (EHDV-6[W]), reducing the titre of either isolate by >5 log_10_ TCID_50_. No significant neutralisation was detected using reference antisera against the other EHDV serotypes, confirming both isolates as EHDV-6. Cross-neutralisation was previously detected in serum neutralisation tests (SNT) between the reference strain of EHDV-6[E] (AUS1981/07) and BAR1983/01, confirming that they both belong to the serotype 6 [15].

### EHDV from Israel 2006

Ten orbivirus isolates were made in embryonated chicken eggs (ECE) from the blood of cows showing clinical signs of disease in Israel during 2006 (ISR2006/01 to ISR2006/09 and ISR2006/13), then grown in BHK-21 cells. RNA extracted from the infected cell pellet was tested using an EHDV group-specific RT-PCR assay (targeting genome Seg-7 – [14], Table S1 - supporting information), generating a cDNA band of the expected size (1162 bp), and providing the first identification of these viruses as EHDV (result not shown). ISR2006/13 was tested by VNT, showing a reduction in titre of 2.5 Log_10_ TCID_50_ with reference antisera to EHDV-7[E] (AUS1981/06), but no significant reduction in titre with reference sera to the other EHDV serotypes. A reduction of >2.0 Log_10_ is usually considered to be indicative of the homologous serotype in this type of assay, confirming the identity of ISR2006/13 as EHDV-7.

cDNA copies were subsequently synthesised from Seg-2 of ISR2006/13, then sequenced using an ‘anchor spacer’ ligated to 3′ termini of the RNA, as described by Maan et al [70]. The upstream terminal 2867 nt/880 aa of Seg-2/VP2 were compared to strains of different EHDV serotypes (Fig. 3), showing highest levels of identity (75% nt/79% aa respectively) with the Australian reference strain of EHDV-7 (AUS1981/06). The detection of EHDV-7 sequences using anchor ligation indicates that EHDV-7 represented at least the dominant component of the virus population in the sample tested.

Recent studies of BTV Seg-2 [73] have detected up to 31% nt variation within a single serotype. Although the levels of nucleotide sequence variation between and within individual EHDV serotypes have not yet been fully explored, initial studies suggest that the same may be true for EHDV [15]. The relationship between Seg-2 of ISR2006/13 and that of other EHDV strains (<30.5% nt identity), is therefore consistent with its serological identification as EHDV-7 (with which it shares 75% nt identity). However, the relationship is quite distant, suggesting that the Australian and Israeli viruses (AUS1981/06 and ISR2006/13) belong to different topotypes (E and W respectively). Seg-2 of ISR2006/13 also showed slightly lower levels of identity with three strains of EHDV-2 (69.3% nt/68.2% aa identity to AUS1979/05; 69.5% nt and 68% aa identity to JAP1959/01; and 67.8% nt/68% aa identity to CAN1962/01). EHDV-2 is the most closely related heterologous serotype to EHDV-7 (Group A - as described by Anthony et al [15], Fig. 3).

**Figure 4:**
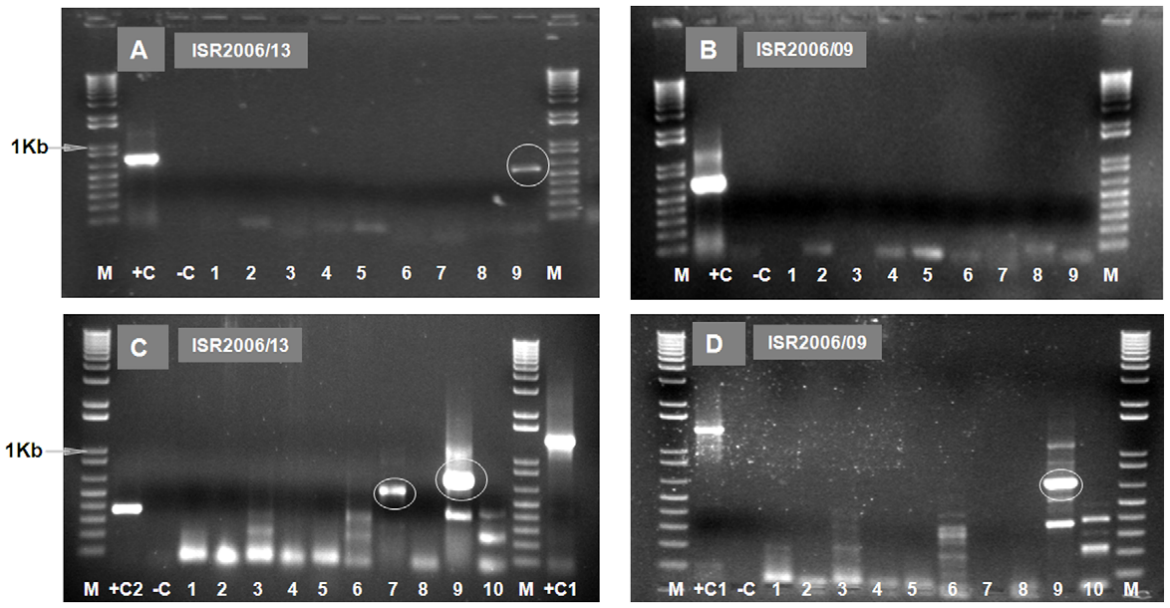
Electrophoretic analysis of cDNA from Seg-2 of EHDV isolates from Israel 2006 using ‘type-specific’ primer-pairs. PCR amplicons were generated from Seg-2 of ISR2006/13 - Panel A and ISR2006/09 - Panel B using primer-pairs ‘1E1’, ‘1W2’, ‘2A1’, ‘4W1’, ‘5E1’, ‘6E1’, ‘7E1’, ‘8E1’, 6W1’ (lanes 1 to 9). A 573 bp cDNA product was obtained with primer-pair ‘6W1’ in ISR2006/13 (lane 9 –Panel A, Table S1 - supporting information) but not in ISR2006/09 (lane 9 – Panel B). Lane M: 1 Kb marker. +C is a positive control using RNA from EHDV-6/AUS1981/07, with primer-pair ‘6A1’ – 664 bp. Lane -C is a negative water control showing no amplification. PCR amplicons were generated from Seg-2 of Israeli isolates (ISR2006/13 –Panel C and ISR2006/09 – Panel D) using primer-pairs ‘1E1’, ‘1W2’, ‘2A1’, ‘4W1’, ‘5E1’, ‘6E1’, 6W1’, ‘7E1’, 7W2’ and ‘8E1’ (lanes 1 to 10 respectively). Expected size cDNA products of 573 and 609 bp were obtained with primer-pairs ‘6W1’ and ‘7W2’ in ISR2006/13 (lanes 7 and 9 – Panel C, Table S1 - supporting information) but only one right sized product of 609 bp was obtained in ISR2006/09 with primer-pair ‘7W2’ (lane 9 – Panel D). Lane M: 1 Kb marker. RNA from BTV-1/RSArrrr/01, with primer-pair ‘1A2’ – 1334 bp and BTV-9/RSArrrr/09, with primer-pair ‘9A2’ - 345 bp were used as positive controls [63] (lane +C1 – Panel D and lane +C2 – Panel C, respectively). Lane -C is a negative water control showing no amplification.

The sequence of Seg-2 from ISR2006/13 was used to design primers ‘7W1’ and ‘7W2’ (see assay development: above). Although cDNAs of the correct size were amplified from Seg-2 of ISR2006/13 RNA using and ‘7W1’ and ‘7W2’, primers for EHDV-6 (‘6W1’ and ‘6W2’) (Table S1 - supporting information) also generated cDNA products of the expected size from this sample (Fig. 2 – Panel C; Fig. 4 - Panel A and C), indicating that it contained both EHDV types 6 and 7. The Seg-2 amplicons generated from ISR2006/13 with primers ‘7W1’ and ‘7W2’ primers gave sequences identical to those obtained by anchor-primer ligation (described above). However, the 573 and 468 bp sequences generated from ISR2006/13 using primer-pairs ‘6W1’ and ‘6W2’s, showed 97% and 98.4% nt sequence similarity to EHDV-6/BAR1983/01, confirming the presence of EHDV-6[W] in the sample. These results also indicate that both EHDV-6[W] and EHDV-7[W] were circulating in Israel during 2006 and correlate with the subsequent detection of EHDV-6 [W] in Turkey during 2007 (see below).

RT-PCR assays using RNA from other Israeli isolates from 2006 (ISR2006/01 to ISR2006/09), generated cDNAs of the correct size with both pairs of EHDV-7 specific primers (‘7W1’ and ‘7W2’) but not with EHDV-6 specific primer-pairs as shown in Fig. 4 – Panel B and D for ISR2006/09. Sequencing of Seg-2 amplicons from these isolates generated identical sequences (to that of ISR2006/13) but showed only 73% nt sequence identity to the reference strains of EHDV-7 (AUS1981/06) confirming the presence of EHDV-7[W] in these Israeli samples.

An antiserum sample from Israeli cattle (from 2006), which neutralised 100 TCID_50_ of the reference strain of EHDV-7[E] (AUS1981/06) at a dilution of 1/320, gave a similar result in SNT with EHDV-2 (the most closely related serotype to EHDV-7). These serological typing results were therefore regarded as inconclusive, but suggest that the animal could previously have been infected with both EHDV-2 and EHDV-7. No results are available for the reactivity of this serum sample with EHDV-6.

### EHDV isolates from the USA 2006

The USA 2006 isolates were initially tested at the Southeastern Cooperative Wildlife Disease Study (SCWDS), USA, for virus neutralisation (VNT) using antisera to EHDV-1 and 2 [17], with negative results. The virus isolates were then tested at USDA National Veterinary Services Laboratories (NVSL) for virus neutralisation using antisera to different EHDV serotypes. A significant reduction in cytopathology was observed in wells inoculated with these viruses and antiserum to EHDV-6 (CSIRO 753). No neutralisation was observed with antisera to EHDV serotypes 1 (previously regarded as type 3), 4, 5, 7 and 8.

**Figure 5:**
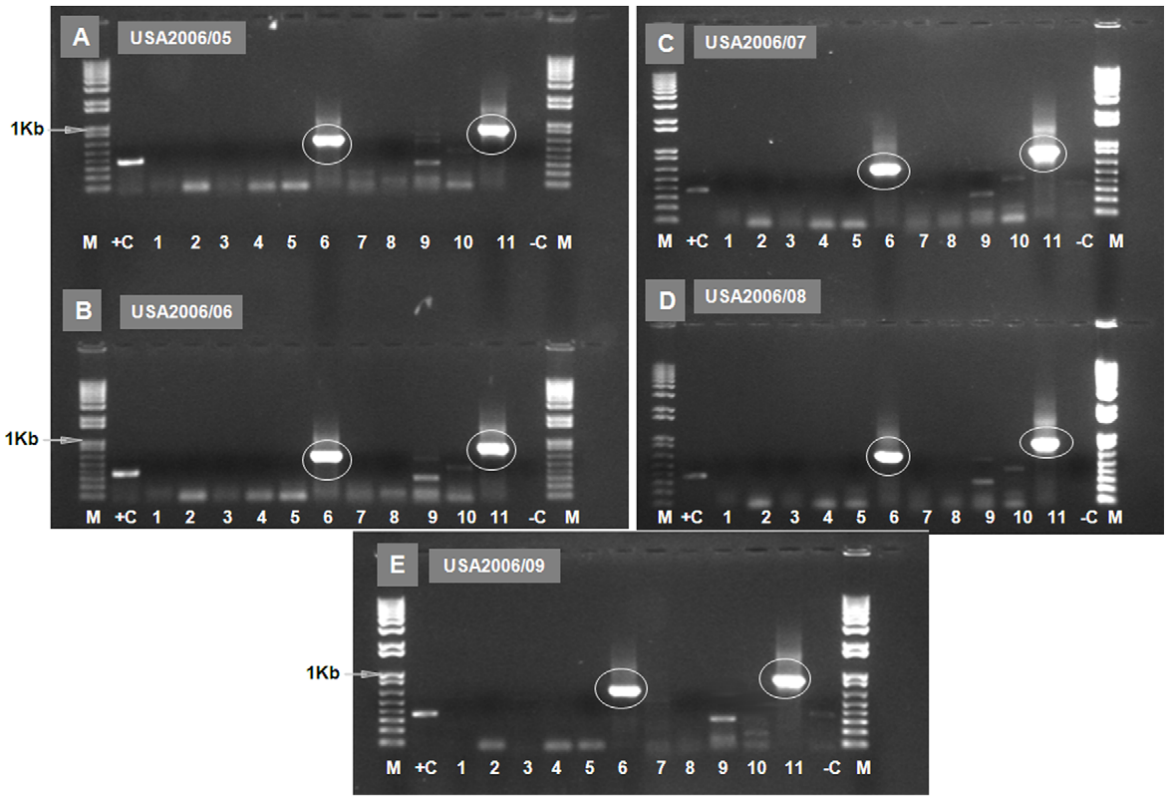
Electrophoretic analysis of cDNA from Seg-2 of EHDV isolates from USA using ‘type-specific’ primer-pairs. PCR amplicons were generated from Seg-2 of isolates from USA, using primer-pairs ‘1E1’, ‘1W2’, ‘2A1’, ‘4W1’, ‘5E1’, ‘6A1’, ‘7E1’, ‘8E1’, ‘6W2’, ‘2A2’ and ‘6A2’ (lanes 1 to 11 respectively - Panels A–E). cDNA products of 664 and 859 bp were obtained with primer-pairs ‘6A1’ and ‘6A2’ in all isolates from USA (USA2006/05, USA2006/06, USA2006/07, USA2006/08 and USA2006/09) (lanes 6 and 11 – Panels A–E, respectively, Table S1 - supporting information). Lane M: 1 Kb marker. RNA from BTV-9/RSArrrr/09 was used as a positive control using primer-pair ‘9A2’ - 345 bp [63] (lane +C). Lane -C is a negative water control showing no amplification.

RNAs extracted from BHK-21 cells infected with five different EHDV isolates from the USA 2006 (USA2006/05 and USA2006/09), were tested with serotype-specific primers directed against Seg-2 of different EHDV serotypes (Table S1 - supporting information). Although strong bands of the expected sizes, were detected with primer-pair ‘6A1’ (664 bp) and ‘6A2’ (859 bp), no amplification was detected with primer-pairs for any of the other EHDV serotypes or topotypes (including primer sets ‘6W1’ and ‘6W2’) (Fig. 5 - Panels A–E). The 664 bp PCR product was sequenced from all five isolates (using ‘6A1’ sequencing primers) and compared to Seg-2 of other EHDV strains (Fig. 3). The highest nt identity level (97.7%) was detected with the Australian reference strain of EHDV-6[E] (AUS1981/07), with lower levels (72.5% identity) to Seg-2 of EHDV-6[W] (BAR1983/01), identifying the serotype and topotype of the isolates as EHDV-6[E]. The level of nt identity in Seg-2 between EHDV-6[E] (AUS1981/07) and 6[W] (BAR1983/01) was 72.9%, while the level between the USA isolates and other serotypes of EHDV, ranged from 39.9%–66.6%.

### EHDV isolates from Turkey 2007

**Figure 6:**
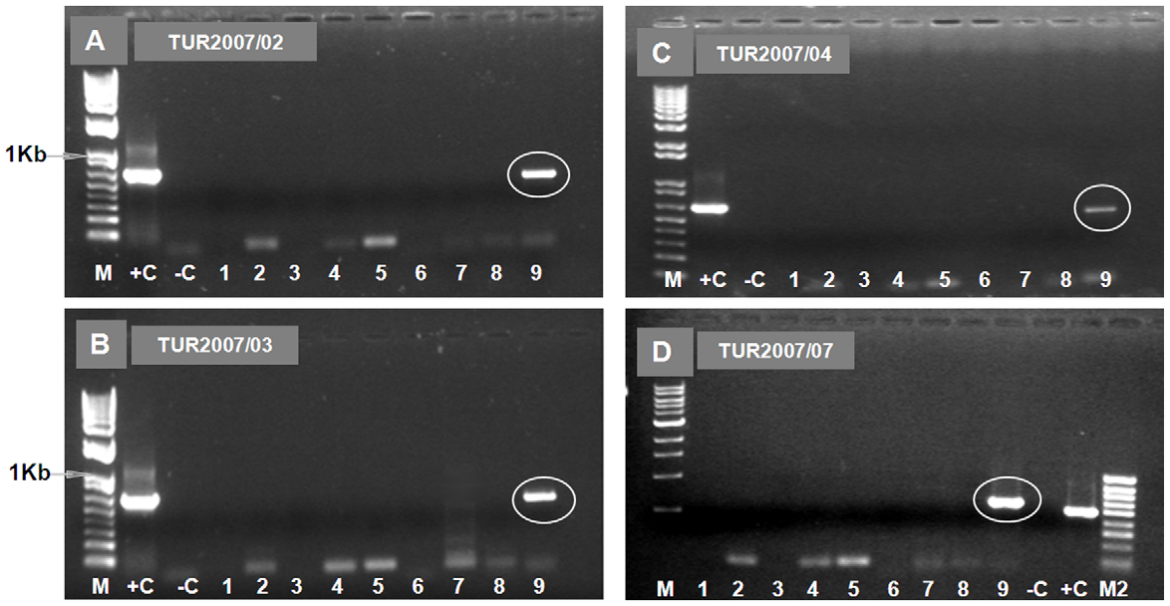
Electrophoretic analysis of cDNA from Seg-2 of EHDV isolates from Turkey using ‘type-specific’ primer-pairs. PCR amplicons were generated from Seg-2 of Turkish 2007 isolates, using primer-pairs ‘1E1’, ‘1W2’, ‘2A1’, ‘4W1’, ‘5E1’, ‘6E1’, ‘7E1’, ‘8E1’, and ‘6W1’ (lanes 1 to 9 respectively - Panels A–D). A cDNA product of 573 bp was obtained with primer-pair ‘6W1’ in all samples from Turkey (TUR2007/02, TUR2007/03, TUR2007/04, and TUR2007/07) (lane 9 – Panels A–D, respectively, Table S1 - supporting information). Lane M: 1 Kb marker. Lane M2: 100 bp marker. RNA from EHDV-6/AUS1981/07, with primer pair ‘6A1’ - 664 bp and EHDV-1/NIG1967/01 with primer-pair ‘1W2’ - 488 bp were used as positive controls (lane +C in Panels A – C and Panel D, respectively). Lane -C is a negative water control showing no amplification.

RNAs extracted from six virus isolates derived from diagnostic blood samples taken from cattle in Turkey during 2007 (TUR2007/01 to TUR2007/06, listed in Table 1) and a bovine lung and spleen tissue homogenate sample (TUR2007/07 - Table 1), were tested by conventional group-specific RT-PCR assays (targeting Seg-7, [14], Table S1 - supporting information). Although these samples were contaminated with bacteria and appeared to be somewhat degraded, a positive result (with a cDNA band of the expected size) was obtained. Real time diagnostic RT-PCR assays for BTV [74], were negative for all of the samples, although group-specific real time RT-PCR assay for EHDV (from LSI) also gave a positive result, with CT values of 20.32–27.5. One serum sample (number A162/07 13) from a different animal also gave a weak +ve result for EHDV specific antibodies by ELISA [75]–[77]. Subsequent RT-PCR assay, using primers ‘6W1’ and ‘6W2’ (Table S1 - supporting information), amplified cDNAs of the appropriate sizes from isolates TUR2007/01, TUR2007/02, TUR2007/03, TUR2007/04, and TUR2007/07, providing the first identification of both the virus type and topotype (Fig. 6 - Panels A–D). No amplification was detected with the other serotype specific primers including ‘6E1’.

Sequencing and phylogenetic analyses of the cDNAs generated (nt 120-684) from TUR2007/02 and TUR2007/07, showed highest levels of nt sequence identity with BAR1983/01 (96.2–96.6%) confirming the virus as EHDV-6[W]. Similarities of Seg-2 from TUR2007/02 and TUR2007/07 with other EHDV serotypes ranged from 43.7% (EHDV-1/NIG1967/01 and EHDV-7/AUS1981/06) to 72.2% (EHDV-8/AUS1982/06), with EHDV-6[E] (AUS1981/07) showing 72.7% identity (Fig. 3). The different isolates of EHDV-6 from Turkey from 2007 showed a minimum level 98.9% nt sequence identity among themselves and 97.3% identity to Seg-2 (120–684 nt) of EHDV-6 ISR2006/13, indicating that they all share a recent common ancestry and suggest they represent continuation of a single outbreak.

## Discussion

Serological ‘typing’ methods (VNT and SNT), based on the specificity of interactions between neutralising antibodies and the outer virion surface (particularly VP2), have been used for many years and with considerable success, as primary diagnostic assays to monitor outbreaks of orbiviral diseases and identify the ‘serotype’ of the virus involved. However, these assays are slow and time consuming (taking weeks), and require access to stocks of well characterised reference antisera and reference virus strains for each serotype [78]. Serological assays are also unable to distinguish individual strains within a single serotype, and are therefore unsuitable for high precision epidemiological studies that need to differentiate closely related virus lineages, or topotypes.

Seg-2 of both EHDV and BTV encodes VP2, the outermost capsid protein and primary serotype-specific antigen of the virus particle [13], [79]–[81]. Seg-2/VP2 are also the most variable genome segment and protein (>45.8% nt and >31.1% aa identity), reflecting the evolution of several distinct serotypes within each *Orbivirus* species/serogroup, and the separation of strains from different geographic origins, into distinct topotypes within these serotypes [2], [9], [15], [78], [79], [82]–[84]. This high degree of variability makes Seg-2/VP2 ideal targets not only for the positive identification of each virus ‘type’ but also for assays or phylogenetic comparisons to distinguish between closely related virus strains [37], [63].

Until recently the limited availability of reference ‘antisera and isolates’ of individual EHDV serotypes and topotypes, have made it difficult to determine the precise serological relationships that exist between different orbivirus strains, by conventional serological methodologies (VNT and SNT assays). Consequently there is some uncertainty in the literature concerning the total number of EHDV serotypes [2], [64], [85]–[87]. Between 8-10 serotypes/strains of EHDV (serotypes 1–8, EHDV-318 and Ibaraki virus (IBAV) have been suggested in the literature [2], although the most recent study by Anthony et al [15] has grouped type 1 and 3 together (as EHDV-1) and strain 318 as a western strain of type 6, making a total of only seven distinct EHDV serotypes.

In contrast 25 serotypes of BTV have been identified by serological assays as well as phylogenetic comparisons of Seg-2/VP2 [63], [73], [88]–[90]. Recently a sequence database for Seg-2 of BTV [73], was used to design oligonucleotide primers and probes for conventional and real-time RT-PCR assays to identify individual BTV types (conventional assays - Mertens et al [63]; in preparation; real-time assays available from LSI).

Anthony et al [15], [91], [92] compared full-genome sequence data for ‘reference’ strains of EHDV, confirming that (like BTV) variations in Seg-2/VP2 correlate with virus serotype. These data have supported the design of EHDV ‘serotype-specific’ primers for use in the RT-PCR assays described here. Multiple primer sets targeting Seg-2 of each EHDV serotype were evaluated, in particular for their ability to identify and discriminate between reference strains or available field isolates of different EHDV serotypes and topotypes, as well as other related orbiviruses, confirming their specificity.

These Seg-2 specific RT-PCR assays and primers, together with associated sequencing studies, allow individual EHDV serotypes (and topotype) to be identified with greater accuracy, sensitivity and more rapidly than by conventional serological typing methods. It is now also possible to identify individual strains and lineages within a single EHDV serotype in a manner that is impossible by conventional serological typing methods, facilitating intense surveillance. These assays can also provide Seg-2 amplicons for sequencing – allowing comparisons to identify serotype, topotype and strain with great precision. Although primer sets were identified that were specific for Seg-2 of each serotype, these vary in their ability to prime cDNA synthesis from different isolates. This reflects high levels of sequence variability in Seg-2 of BTV and related orbiviruses, even within a single serotype, depending largely on the geographical origin (topotype) of the isolate [15], [36].

In recent years (2006–2007), several EHD outbreaks caused by different serotypes that have occurred in different locations around the world, have been identified using the type-specific assays described here. These include EHDV-6[W] recently recognized as an emerging pathogen of cattle in Algeria, Morocco (in 2006) and Turkey (2007) [34]. EHDV-7[W] was also identified in Israel in 2006 [35]. EHDV isolates from white-tailed deer in Indiana and Illinois [17], were confirmed as type 6 and for the first time identified as an eastern topotype of type 6 (EHDV-6[E]), a serotype and topotype originally isolated in Australia. Genetic characterization of EHDV-6[E] Indiana (304-06) [17] showed that it is a reassortant virus, with outer capsid proteins that determine serotype (VP2 and VP5) derived from an exotic strain of EHDV-6[E], although how this strain arrived in the USA remains uncertain. The remaining structural and non-structural proteins are derived from an indigenous strain of EHDV-2 (Alberta), making it difficult to distinguish this strain from other American strains of EHDV using group specific serological or RNA sequence based assays (RT-PCR) that target these more conserved genome segments and proteins.

These studies highlight a need to establish reference strains for both eastern and western topotypes of each EHDV serotype, with associated sequence data for Seg-2 (as listed in Table 1; [48]). The new EHDV ‘type-specific’ assay systems described here provide a valuable tool for future studies, to provide more information concerning the persistence and movements of individual virus strains in the field. The assays were designed based on sequence data from a limited pool of available EHDV strains, primarily to distinguish between isolates of the different EHDV serotypes, and the resulting assays are successful in this respect. However, it is possible that EHDV strains will be identified in the future that cross-react by RT-PCR assay with heterologous serotypes, or fail to amplify using these type-specific primers. This will require further sequence analyses/comparison and primer redesign to maintain the efficacy and specificity of these assays. The fine-scale differentiation between individual EHDV strains within each serotype and topotype depends on the availability and characterisation of well documented isolates, such as those stored and maintained in the reference collection at IAH Pirbright [48], [49].

Conventional and real-time RT-PCR assays will inevitably increase the speed and reliability of laboratory-based diagnostic testing for EHDV. It is not easy to compare the sensitivity and specificity of type-specific and group-specific, conventional and real-time RT-PCR assays, targeting different orbivirus genome segments, in a meaningful way. However, in our experience of routine detection of BTV and EHDV RNA in diagnostic tissue samples, or cell-culture materials [14], [64]–[68], [74], those samples that give CT values of <30 by real-time PCR (equivalent to ∼100 copies – data supplied by LSI for their Seg-9 based EHDV group-specific assay), will also reliably generate cDNA amplicons by conventional RT-PCR assay targeting the appropriate Seg-2 [63]. The serotype specific assays described here can be used to detect and ‘type’ individual strains of EHDV in the absence of reference antisera for each serotype. Unlike real-time RT-PCR assays, the conventional type-specific primers can also be used to sequence any of the cDNA amplicons that are generated, providing further and definitive proof of the relationships between individual virus strains in Seg-2. Phylogenetic comparisons of the resulting EHDV Seg-2 sequences will help determine the worldwide prevalence and distribution of different EHDV serotypes and topotypes, improving our understanding of the epidemiology and transmission of the virus. The data generated will inform the design and implementation of future control strategies, including vaccination.

## Supporting Information

Table S1 Primers for specific amplification of Seg-2 from various EHDV serotypes in RT-PCR assays.
(0.11 MB DOC)
