# Supplementary material for: RT-PCR Assays for Seven Serotypes of Epizootic Haemorrhagic Disease Virus & Their Use to Type Strains from the Mediterranean Region and North America
Source: PLoS One. 2010 Sep 17;5(9):e12782. doi: 10.1371/journal.pone.0012782 (PMC2941451; doi:10.1371/journal.pone.0012782)
Supplement: Table S1 — Primers for specific amplification of Seg-2 from various EHDV serotypes in RT-PCR assays. (0.11 MB DOC) [file pone.0012782.s001.doc]

**Table S1: Primers for specific amplification of Seg-2 from various EHDV serotypes in RT-PCR assays.**

|  | **Primer Name**** | **Primer Pair*** | **Position on genome Seg-2 (nt)** | **Predicted Product size (bp)** | **IAH-P*** dsRNA virus reference collection number of the strain from which primer were designed (Accession number of Seg-2)** |
| --- | --- | --- | --- | --- | --- |
| **EHDV type specific primers targeted against genome segment 2** | | | | | |
| 1E1 | EHDV-1/S2/103-124F | ATATCCTGGCGGAACCATATGG | 103-124 | 918 (Fig. 1 – Panel A) | AUS1995/02 (HM156728) |
|  | EHDV-1/S2/1021-1001R | ATCTGCCTGATGTGGTGTTTG | 1021-1001 |  |  |
| 1E2 | EHDV-1/S2/1324-1344F | TGATGTGAAAATTCACGCTCG | 1324-1344 | 660 (Fig. 1 – Panel A) | AUS1995/02 (HM156728) |
|  | EHDV-1/S2/1984-1964R | GCTAGACAGCTCAAGTATCCT | 1984-1964 |  |  |
| 1W1 | EHDV-1/S2/1828-1851F | CAAGGAAATATATGACGATTTTAT | 1828-1851 | 888 (Fig. 1 – Panel B) | USA1955/01 (AM744978) |
|  | EHDV-1/S2/2716-2689R | GTCGTCCTCGTTTAACGCTCCCATTCCG | 2716-2689 |  |  |
| 1W2 | EHDV-1/S2/1828-1851F | TGAGGAAATATATGATGAATTCAC | 1828-1851 | 488 (Fig. 1 – Panel B) | NIG1967/01 (AM745008) |
|  | EHDV-1/S2/2595-2316R | GAATTATCTGTGCAAGGATCTG | 2187-2171 |  |  |
| 1W3 | EHDV-1/S2/286-310F | TGAGGTTGARTTAGARAATCAGCTG | 286-310 | 735 (Fig. 1 – Panel B) | USA1955/01 (AM744978); NIG1967/01 (AM745008) |
|  | EHDV-1/S2/1021-1005R | YTCCGGTTGATCTCGAG | 1021-1005 |  |  |
| 1A1 | EHDV-1/S2/125-147F | GARCCGCATCAAATGTATGAYMG | 125-147 | 1199 (Fig. 1 – Panel A) | USA1955/01 (AM744978); NIG1967/01 (AM745008) and AUS1995/02 (HM156728) |
|  | EHDV-1/S2/1324-1302R | RYKYTTATCCCATTCYGWYAYMA | 1324-1302 |  |  |
| 1A2 | EHDV-1/S2/363-384F | GAAARYTRCAGWTRGAATTGAG | 363-384 | 1500 (Fig. 1 – Panel A) | USA1955/01 (AM744978); NIG1967/01 (AM745008) and AUS1995/02 (HM156728) |
|  | EHDV-1/S2/1863-1839R | TCTYGYAYCGGTDTRAAWTCDTCAT | 1863-1839 |  |  |
| 2E1 | EHDV-2/S2/284-306F | GTTCTCAAGGAATCGAAATACAG | 284-306 | 2509 (data not shown) | AUS1979/05 (AM744988); JAP1959/01 (AM745078) |
|  | EHDV-2/S2/2793-2771R | GCGCATGTACATATTGTTCAACA | 2793-2771 |  |  |
| 2W1 | EHDV-2/S2/299-317F | GAGCGTGGAAAGAAGTAAT | 299-317 | 1942 (Fig. 1 – Panel D) | CAN1962/01 (AM744998) |
|  | EHDV-2/S2/2241-2219R | TTGAGAAATTTGGAGCTAGTATG | 2241-2219 |  |  |
| 2A1 | EHDV-2/S2/1644-1664F | TTTAARRTAAGACGGGTCGAG | 1644-1664 | 531 (Fig. 1 – Panel C) | CAN1962/01 (AM744998); AUS1979/05 (AM744988); JAP1959/01 (AM745078) |
|  | EHDV-2/S2/2175-2151R | TYAY CACWCGGATCTCCTCYTCGCT | 2175-2151 |  |  |
| 2A2 | EHDV-2/S2/113-133F | GAAARRYGARRRTAGATTGCC | 113-133 | 612 (Fig. 1 – Panel C) | CAN1962/01 (AM744998); AUS1979/05 (AM744988); JAP1959/01 (AM745078) |
|  | EHDV-2/S2/745-718R | TSATCTGGGYTYATACTWGTRGTYAGYG | 745-718 |  |  |
| 4W1 | EHDV-4/S2/294-317F | TAAAGGTGGAATAGGTGCCGGAAG | 294-317 | 1161 (Fig. 2 – Panel A1) | NIG1968/01 (AM745018) |
|  | EHDV-4/S2/1455-1432R | TATGCTGTGTACTATTACATCTGG | 1455-1432 |  |  |
| 4W2 | EHDV-4/S2/1863-1883F | TATCAAGAAGGATGAGCAGCT | 1863-1883 | 428 (Fig. 2 – Panel A1) | NIG1968/01 (AM745018) |
|  | EHDV-4/S2/2291-2271R | GCTAATGGTACTTTCTTGAAG | 2291-2271 |  |  |
| 5E1 | EHDV-5/S2/297-318F | TACCACACGAGAGAAGGAGGGT | 297-318 | 680 (Fig. 2 – Panel A2) | AUS1977/01 (AM745028) |
|  | EHDV-5/S2/977-960R | GAATCCATGGCATCCGAG | 977-960 |  |  |
| 5E2 | EHDV-5/S2/387-408F | GACCGGAGTAGATTATACGAAT | 387-408 | 2199 (Fig. 2 – Panel A2) | AUS1977/01 (AM745028) |
|  | EHDV-5/S2/2586-2570R | GACCATGTCACACGCCATTG | 2586-2570 |  |  |
| 6E1 | EHDV-6/S2/168-190F | TAACACAAGGAGAAGCCCGTAAT | 168-190 | 1772 (data not shown) | AUS1981/07 (AM745038) |
|  | EHDV-6/S2/1940-1918R | TTGAGAAAACTCGGGTAAATAAT | 1940-1918 |  |  |
| 6W1 | EHDV-6/S2/107-129F | GCTTATAGTGGTGAAAAGGAAGG | 107-129 | 573 (Fig. 2 – Panel B) | BAR1983/01 (AM745068); ALG2006/02 (HM156729)  MOR2006/05 (HM156730) |
|  | EHDV-6/S2/680-654R | GTGTACCAATCGGTATCATCTGTCTTG | 680-654 |  |  |
| 6W2 | EHDV-6/S2/1855-1871F | GGAGGAAGGTGGAGAGG | 1855-1871 | 468 (Fig. 2 – Panel B) | BAR1983/01 (AM745068) |
|  | EHDV-6/S2/2323-2302R | GCTATCCATCGTTTGGTGTTTG | 2323-2302 |  |  |
| 6A1 | EHDV-6/S2/297-317F | GTGTGATACTATTAGAACACG | 297-317 | 664 (Fig. 2 – Panel B) | AUS1981/07 (AM745038) |
|  | EHDV-6/S2/961-942R | GTAGTCCGCACTCGTTCCAG | 961-942 |  |  |
| 6A2 | EHDV-6/S2/1856-1877F | GATGAAGATGGTGAGAATGGTG | 1856-1877 | 859 (Fig. 2 – Panel B) | AUS1981/07 (AM745038) |
|  | EHDV-6/S2/2715-2695R | GCCTCTGCGTTGCTGTTAGCG | 2715-2695 |  |  |
| 7E1 | EHDV-7/S2/299-318F | GGCAAAAGGTGAAGAACAGG | 299-318 | 700 (Fig. 2 – Panel C) | AUS1981/06 (AM745048) |
|  | EHDV-7/S2/999-978R | GCAAGTTGATAACCAACTGCTG | 999-978 |  |  |
| 7E2 | EHDV-7/S2/1857-1877F | GATGAAAATGTTGTAAGAAGG | 1857-1877 | 730 (Fig. 2 – Panel C) | AUS1981/06 (AM745048) |
|  | EHDV-7/S2/2587-2564R | GGATCAATTATTTCTTTTCGTTTG | 2587-2564 |  |  |
| 7W1 | EHDV-7/S2/300-318F | GGGAAGTGGCGGACAAG | 300-318 | 1978 (Fig. 2 – Panel C) | ISR2006/13 (HM156731) |
|  | EHDV-7/S2/2278-2262R | GCCGTCTCAGAAGGACG | 2278-2262 |  |  |
| 7W2 | EHDV-7/S2/1669-1686F | GGCAGAACAAAGGTCAAG | 1669-1686 | 609 (Fig. 2 – Panel C) | ISR2006/13 (HM156731) |
|  | EHDV-7/S2/2278-2262R | GCCGTCTCAGAAGGACG | 2278-2262 |  |  |
| 8E1 | EHDV-8/S2/101-123F | GTTAGGACTTACAATGACGAATT | 101-123 | 803 (Fig. 2 – Panel D) | AUS1982/06 (AM745058) |
|  | EHDV-8/S2/904-880R | GATATTTCTTCGATCCGCTCACTG | 904-880 |  |  |
| 8E2 | EHDV-8/S2/1796-1816F | GAGTCGATAAGCAACACAAAG | 1796-1816 | 772 (Fig. 2 – Panel D) | AUS1982/06 (AM745058) |
|  | EHDV-8/S2/2563-2543R | GCTGATGTGTCTGATTGTATG | 2563-2543 |  |  |
| **EHDV group specific primers targeted against genome segment 7** | | | | | |
| EHDV/S7/  Group | EHDV/S-7/1-21F | GTTAAAATTTGGTGAAGATGG | 1-27 | 1162 (data not shown) | AM744983, AM745003, AM745013, AM745023, AM745073, AM744993, AM745033, AM745043, AM745053, AM745063, AM745083 |
|  | EHDV/S-7/1162-1140R | GTAAGTTGAATTTGGGAAGACG | 1162-1140 |  |  |

*Primer-pairs (identified by the letter A, indicating ‘all’) were selected to detect both eastern and western isolates of each EHDV serotype. Other primer-pairs identified by the letters ‘E’ and ‘W’ are targeted to amplify Seg-2 of either eastern or western topotypes of each serotype, respectively.

**Individual primers are identified by the EHDV serotype (e.g. EHDV-1) followed by the S2 (to indicate Seg-2), then a range of numbers to indicate the relative start and end nucleotide position of the primer within segment 2, followed by F or R to indicate forward or reverse orientation.

***Institute for Animal Health, Pirbright.
